# Arrested Substrate Binding Resolves Catalytic Intermediates in Higher‐Plant Water Oxidation

**DOI:** 10.1002/anie.202012304

**Published:** 2020-12-10

**Authors:** Georgia Zahariou, Nikolaos Ioannidis, Yiannis Sanakis, Dimitrios A. Pantazis

**Affiliations:** ^1^ Institute of Nanoscience & Nanotechnology, NCSR “Demokritos” Athens 15310 Greece; ^2^ Max-Planck-Institut für Kohlenforschung Kaiser-Wilhelm-Platz 1 45470 Mülheim an der Ruhr Germany

**Keywords:** bioinorganic chemistry, catalysis, EPR spectroscopy, photosynthesis, water oxidation

## Abstract

Among the intermediate catalytic steps of the water‐oxidizing Mn_4_CaO_5_ cluster of photosystem II (PSII), the final metastable S_3_ state is critically important because it binds one substrate and precedes O_2_ evolution. Herein, we combine X‐ and Q‐band EPR experiments on native and methanol‐treated PSII of *Spinacia oleracea* and show that methanol‐treated PSII preparations of the S_3_ state correspond to a previously uncharacterized high‐spin (*S=*6) species. This is confirmed as a major component also in intact photosynthetic membranes, coexisting with the previously known intermediate‐spin conformation (*S=*3). The high‐spin intermediate is assigned to a water‐unbound form, with a Mn^IV^_3_ subunit interacting ferromagnetically via anisotropic exchange with a coordinatively unsaturated Mn^IV^ ion. These results resolve and define the structural heterogeneity of the S_3_ state, providing constraints on the S_3_ to S_4_ transition, on substrate identity and delivery pathways, and on the mechanism of O−O bond formation.

## Graphical abstract

Electron paramagnetic resonance spectroscopy reveals that the final metastable catalytic state (S_3_) in biological water oxidation in higher plants is a mixture of a high‐spin and an intermediate‐spin form. These two forms represent distinct structural components related to water binding and activation on the path towards oxygen evolution.

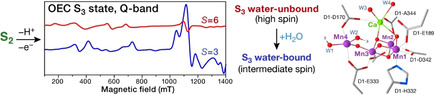

## Introduction

**Figure 1:**
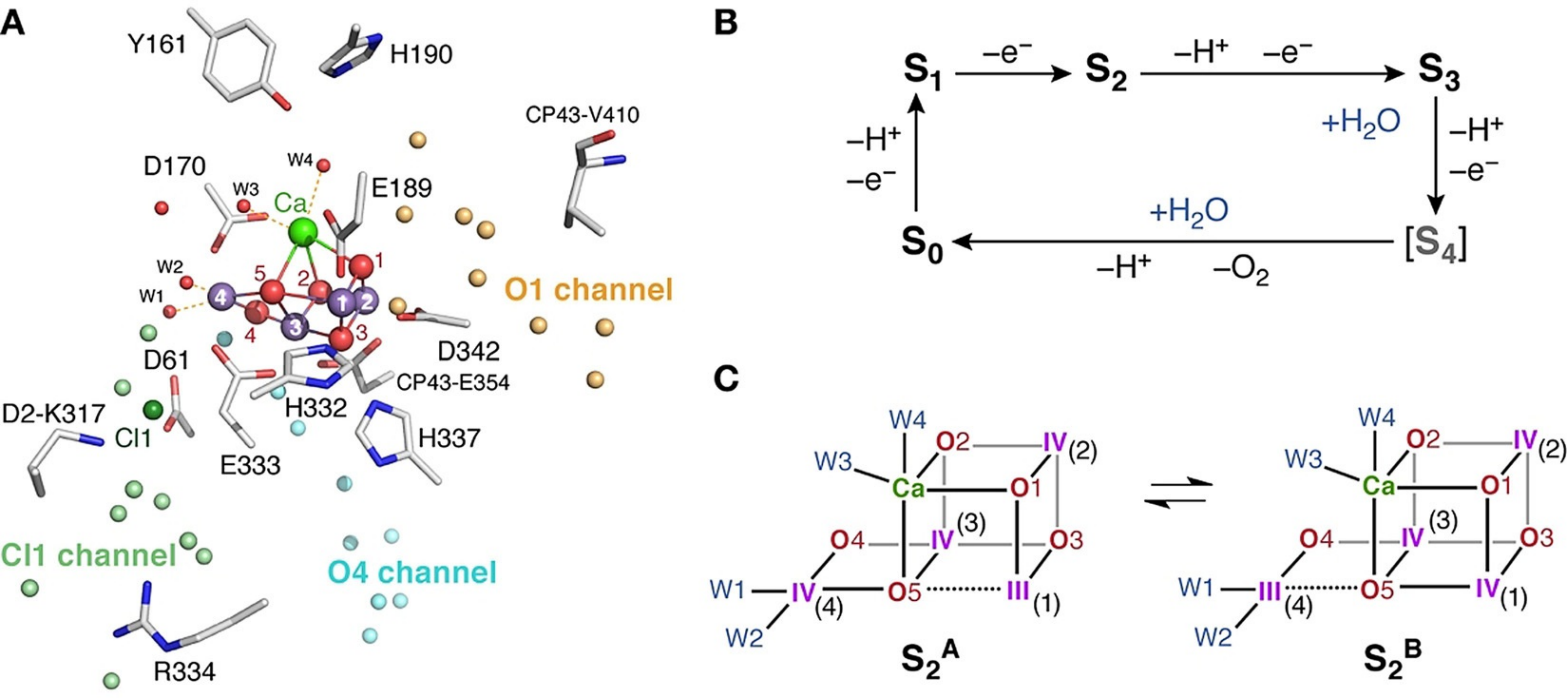
A) At least three water channels identifiable around the Mn_4_CaO_5_ OEC cluster from available crystallographic models of PSII have been discussed as likely access pathways for methanol in higher plants. A close approach to Mn4, for example via the O4 channel, is uniformly favored by hyperfine sublevel‐ correlation (HYSCORE), electron nuclear double resonance (ENDOR), and computational studies.[^9^, ^10^, ^11^, ^12^] B) Cycle showing the S‐states of the OEC cluster. Two water substrates are bound at temporally unresolved intermediates between the S_2_ and S_0_ steps of the cycle. C) Schematic depiction of the cluster conformation in the S_2_ state. Four water or hydroxide ligands (W1‐W4) are attached to Ca and Mn4. EPR spectroscopy reveals two valence isomers in the S_2_ state that differ in Mn oxidation states.[^16^, ^17^, ^18^] The Mn^III^ ion in any given isomer has a formally available site for water coordination in the S_3_ state. The isomer on the left with the +III ion at Mn1 (S_2_^A^) gives rise to the multiline *g=*2 (*S=*1/2) EPR signal, whereas the one on the right with the +III ion at Mn4[^19^, ^20^] (S_2_^B^) gives rise to low‐field *g*≥4 (*S*≥5/2) signals.

Photosystem II (PSII) catalyzes the biologically fundamental reaction of light‐driven oxygen evolution from water. The active site of water oxidation, the Oxygen Evolving Complex (OEC), contains an inorganic Mn_4_O_5_Ca cluster whose catalytic cycle involves four light‐driven oxidation steps denoted as S_0_→S_1_, S_1_→S_2_, S_2_→S_3_, and S_3_→[S_4_]→S_0_, accompanied by progressive removal of four protons from two bound water molecules (Figure 1). O−O bond formation and O_2_ evolution occurs during the transition from the last metastable S_3_ state to S_0_ via an experimentally unresolved mechanistic sequence. Given that one of the two substrate waters is assumed to bind upon completion of the S_2_→S_3_ transition and that the S_3_ state directly precedes O_2_ evolution, it has been the target of intense efforts to elucidate its geometric and electronic structure. Recent crystallographic studies of cyanobacterial PSII that employ femtosecond X‐ray free electron laser (XFEL) pulses support the inclusion of a sixth oxygen ligand in the S_3_ state,[^1^, ^2^, ^3^] as postulated by prior experimental studies.[^4^, ^5^, ^6^] However, the various crystallographic models for the S_3_ state are inconclusive with respect to the precise geometry of the cluster, mutually incompatible with respect to the implied electronic structure,[^1^, ^2^, ^3^] for example, peroxo^[1]^ versus oxyl^[3]^ formation, and seemingly inconsistent with spectroscopic studies that assign an all‐Mn^IV^ cluster to the S_3_ state.[^6^, ^7^, ^8^] Beyond the single‐component picture derived from averaging structural methods, electron paramagnetic resonance (EPR) studies document the presence of multiple, EPR‐active and EPR‐inactive, S_3_ populations at X‐band frequencies (≈9.5 GHz). An important fact utilized in the present work is that the spectroscopic phenomenology is species‐dependent, suggesting that different components of the S_3_ state can be accessed more easily in specific photosynthetic organisms.

Key questions in biological water oxidation concern the precise cascade of transformations leading to the S_3_ state, the composition of the latter, the details of water binding, and the conformation of the inorganic cluster that is active in dioxygen evolution. To address these questions, it is necessary to resolve distinct intermediates experimentally, which can be accomplished by slowing down S‐state transitions and enhancing distinct S‐state components. One way of achieving this is by using a water substrate analogue sufficiently small to reach the active site via physiological water channels yet sufficiently large to hinder water delivery through that channel. It should also be non‐coordinating so that the geometric and electronic structure of the OEC is not fundamentally altered. These conditions are fulfilled by methanol, which accesses the OEC of higher plants up to the terminal point of at least one water channel.[^9^, ^10^, ^11^] Differences in channel architecture between organisms are consistent with the ability of methanol to access the site of water oxidation in higher plants,[^12^, ^13^] whereas it interacts only remotely with the cyanobacterial OEC,[^14^, ^15^] dictating the use of plant PSII for these experiments.

Here we report combined EPR studies at X‐ and Q‐band in intact spinach (*S. oleracea*) PSII membranes and in spinach PSII preparations in the presence of 5 % MeOH. Our results successfully resolve a previously unidentified high‐spin (*S=*6)/ intermediate‐spin (*S=*3) heterogeneity in the S_3_ state. Crucially, the high‐spin component is found to represent the majority constituent of the S_3_ state, necessitating reappraisal of current ideas about the composition of this state and the mechanism of water oxidation. The high‐spin population is assigned to the catalytically active conformation of the OEC that is able to bind substrate water at a coordinatively unsaturated Mn^IV^ ion or possibly progress directly to the final oxygen‐evolving stage of the cycle.

## Results and Discussion

### S_3_‐State X‐ and Q‐Band EPR of Native and MeOH‐Treated Spinach PSII

**Figure 2:**
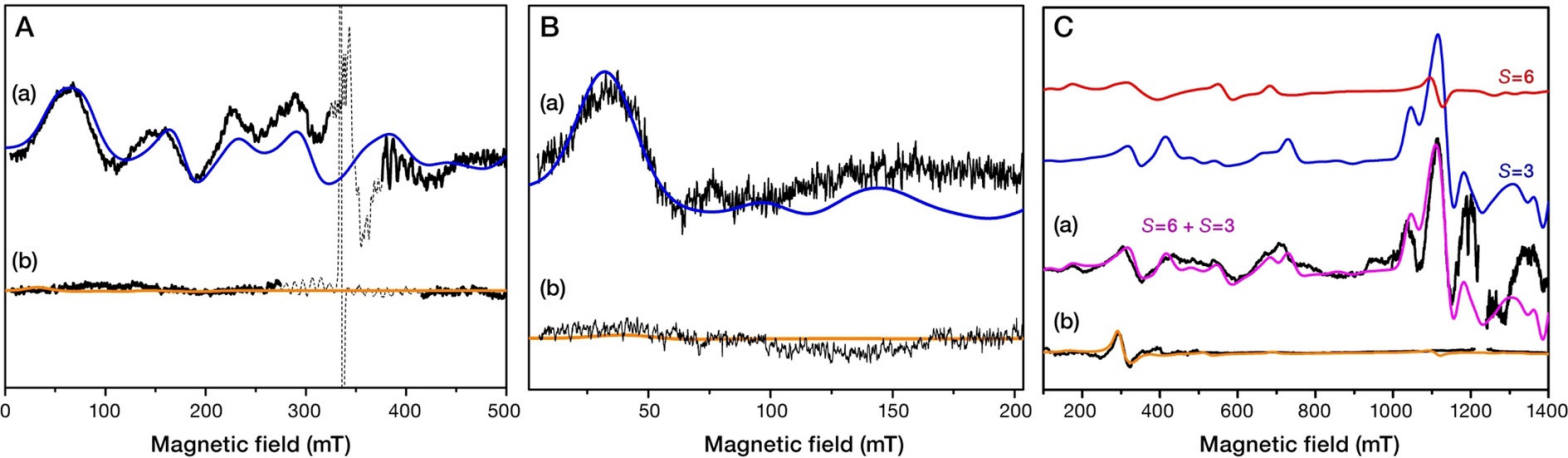
A) Experimental EPR spectra at X‐band in perpendicular mode (black curves) in intact PSII (trace a) and in MeOH containing PSII (trace b), together with their simulation curves obtained by using *S=*3, *g=*2, *D*=0.179 cm^−1^, *E*/*D*=0.28, linewidth=30 mT (blue curve) and *S=*6, *g=*1.98, *D*=1.523 cm^−1^, *E*/*D*=0.14 (orange curve). In order to account for the line shape, a Gaussian distribution on the parameter *D* was assumed with a width of *σ_D_*=0.018 cm^−1^ for the *S=*3 configuration. The dotted line represents the contribution of Chl*z* ^+^ species. B) Experimental EPR spectra at X‐band in parallel mode (black curves) in intact PSII (trace a) and in MeOH containing PSII (trace b), with their simulation curves (blue and orange curves) obtained by the same respective parameters as in (A). C) Experimental EPR spectra at Q‐band in perpendicular mode (black curves) in intact PSII (trace a) and in MeOH containing PSII (trace b), together with their theoretical curves. The S_3_ simulated spectrum for the intact PSII (magenta curve) is obtained by the sum of two theoretical curves arising by using *S=*3, *g=*2, |*D*|=0.179 cm^−1^, *E*/*D*=0.28, σ_*D*_=0.018 cm^−1^, linewidth=30 mT (blue curve), and *S=*6, *g=*2, *D*=+1.51 cm^−1^, σ_*D*_=0.017 cm^−1^, *E*/*D*=0.138, linewidth=30 mT (red curve). The S_3_ simulated spectrum of the MeOH containing PSII (orange curve) originates by using *S=*6, *g=*1.98, *D*=+1.523 cm^−1^, σ_*D*_=0 cm^−1^, *E*/*D*=0.14, with a linewidth of 18 mT.

The S_3_ EPR spectra in intact PSII and in MeOH‐containing PSII preparations from spinach at X‐ and Q‐band together with their simulation curves are shown in Figure 2. In untreated samples the X‐band spectra at both perpendicular and parallel modes can be successfully described by the spin Hamiltonian parameters *S=*3, *g=*2, |*D*|=0.179 cm^−1^, and *E*/*D*=0.28, similar to those reported previously.[^21^, ^22^] This signal is attributed to an all‐Mn^IV^ form of the OEC, with all Mn ions being electronically similar and six‐coordinate.^[6]^ The present results are therefore consistent with prior spectroscopic studies and with the concept that Mn oxidation takes place in all S‐state transitions, in contrast to the hypotheses of early‐stage substrate oxidation or O−O bond formation advanced by certain interpretations of XFEL data.[^1^, ^2^, ^3^, ^23^, ^24^, ^25^, ^26^] Regarding the S_3_ Q‐band EPR spectrum of intact PSII, the theoretical curve obtained by assuming the aforementioned spin Hamiltonian parameters fits the experimental EPR signals mostly at the high field region (*g*_eff_≈2). However, several features centered at lower magnetic fields do not match the corresponding areas of the theoretical curve (see Supporting Information, Figure S1). This indicates the presence of an additional spin configuration of the S_3_ state that is “EPR silent” at X‐band.

S_3_ EPR measurements at Q‐band in 5 % MeOH‐treated preparations show that the features attributed to the *S=*3 signal practically disappear and only one low field EPR derivative at *g*_eff_≈8 is observed, that is very similar to that at the corresponding region in untreated PSII preparations (Figure 2 C). The recently reported spin Hamiltonian parameters that describe the S_3_ experimental spectra in methanol containing cyanobacterial PSII^[15]^ cannot reproduce the present S_3_ experimental spectra in spinach PSII. Detailed simulations show that among all possible integer spin values (*S=*1–6) that may originate from the exchange couplings within the OEC in the S_3_ state, the highest possible spin of *S=*6 provides by far the best match for the experimental low‐field feature (see Supporting Information, Figure S2). The parameters of *D*=+1.523 cm^−1^ and *E*/*D*=0.14 for the *S=*6 configuration uniquely describe the experimental spectrum at Q‐band, since the position of the low field derivative feature is very sensitive to even small changes of the *D* and *E*/*D* parameters from the above values. Additionally, with the aforementioned parameters, the theoretical spectrum at X‐band presents, correctly, no EPR signal, as required by the experimental data (curves (b) of Panels A and B of Figure 2) and in line with previous reports for MeOH‐containing PSII preparations.^[27]^ The results show that the set of spin Hamiltonian parameters used for the simulation of the *S=*6 signal uniquely characterize the S_3_ state in MeOH‐treated spinach PSII.

Owing to the close similarity of the Q‐band EPR features around *g*_eff_≈8 of the S_3_ state in both intact and MeOH‐containing PSII preparations, the EPR signal in untreated preparations that do not match the *S=*3 configuration can be described with spin Hamiltonian parameters quite similar to those for MeOH‐treated PSII. As shown in Panel C of Figure 2, the sum of the two simulated curves obtained by assuming spin Hamiltonian parameters of *S=*3, *g=*2, |*D*|=0.179 cm^−1^, *E*/*D*=0.28 and *S=*6, *g=*2, *D*=+1.51 cm^−1^, *E*/*D*=0.138 satisfactorily reproduces the complete S_3_ experimental spectrum at Q‐band (see also Figure S3).

These observations strongly indicate that two spin configurations of *S=*3 and *S=*6 coexist physiologically in the S_3_ oxidation state in intact spinach PSII membranes. By taking into account the relative intensities of the *S=*3 and *S=*6 simulated curves, as well as the thermal occupation of the respective ground states *S=*6 and *S=*3 multiplets (see Figures S4 and S5), we estimate ca. 20 % for the *S=*3 and 80 % for the *S=*6 configuration. We assign the latter to the S_3_ population referred to in the past as an EPR‐inactive S_3_ form.^[28]^ With these values for the zero field splitting parameters no X‐band signals are expected. The precise factors that determine the relative populations of these states under physiological conditions remain under investigation.

### Structural Interpretation and Role of Anisotropic Exchange

**Figure 3:**
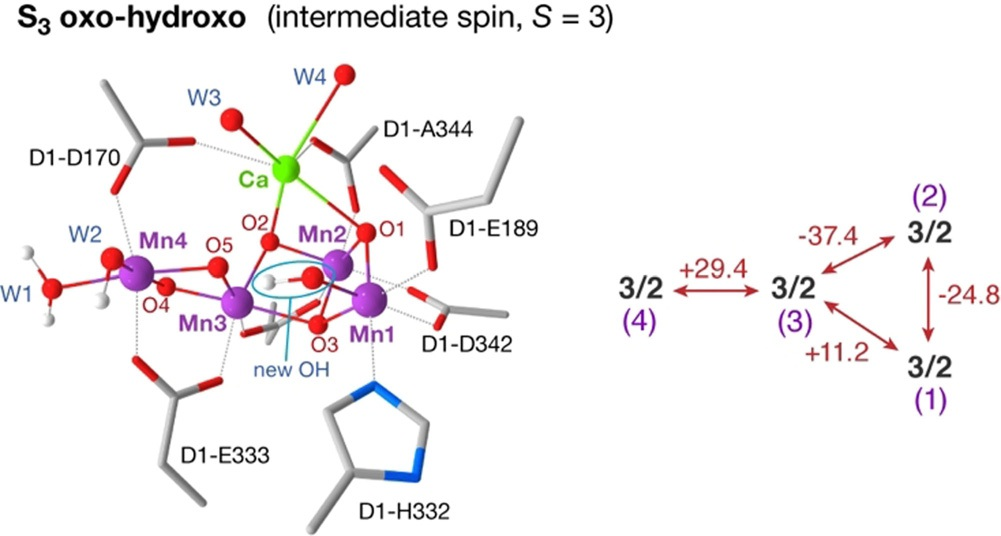
Structural interpretation of the *S=*3 form of the S_3_ state. This “oxo‐hydroxo” conformation bears an additional hydroxy ligand on the Mn1 ion, labelled “new OH”, compared to the main conformation of the cluster in the S_2_ state, rendering all Mn^IV^ centers 6‐coordinate and quasi‐isotropic.^[6]^ Although the precise arrangement and protonation state of the additional Mn1 ligand remains under discussion, the computational model depicted here satisfies the EPR observations regarding the *S=*3 component of the S_3_ state. The “new OH” may originate from direct insertion at Mn1 or from reorganization of the cluster following initial water binding at another Mn center. The accompanying Scheme depicts computed pairwise exchange coupling constants reported by Cox et al.^[6]^ (see also Figure S4; the original values were converted to conform with our convention for the Heisenberg exchange Hamiltonian, *H*=+Σ*J_ij_S_i_S_j_*, where negative *J* values denote ferromagnetic interaction).

Integer spin state EPR signals, initially attributed to *S=*1 by X‐band EPR spectroscopy[^27^, ^29^] and later revised to *S=*3 by Q‐band experiments,^[21]^ have long been associated with the S_3_ state. A commonly accepted geometric conformation of the OEC cluster that satisfies the spectroscopic requirements of an *S=*3 ground state and for all Mn centers being isotropic octahedrally coordinated Mn^IV^ ions, as indicated by electron‐electron double resonance (ELDOR) detected nuclear magnetic resonance experiments (EDNMR),^[6]^ is an “oxo‐hydroxo” conformation (Figure 3), where a water‐derived OH ligand completes the coordination sphere of Mn1 compared to the dominant isomeric conformation of the preceding S_2_ state. Modified spectral forms of the *S=*3 EPR signal have been reported in cation (Ca^2+^/Sr^2+^) or anion (Cl^−^/I^−^) substituted and MeOH‐treated cyanobacterial PSII.[^15^, ^28^] By contrast, the *S=*6 state described in the present work for spinach PSII is experimentally identified for the first time, despite representing apparently the majority species. Its drastically different spectroscopic properties are indicative of fundamental differences in the electronic and geometric structure of the cluster compared to the *S=*3 form.

**Figure 4:**
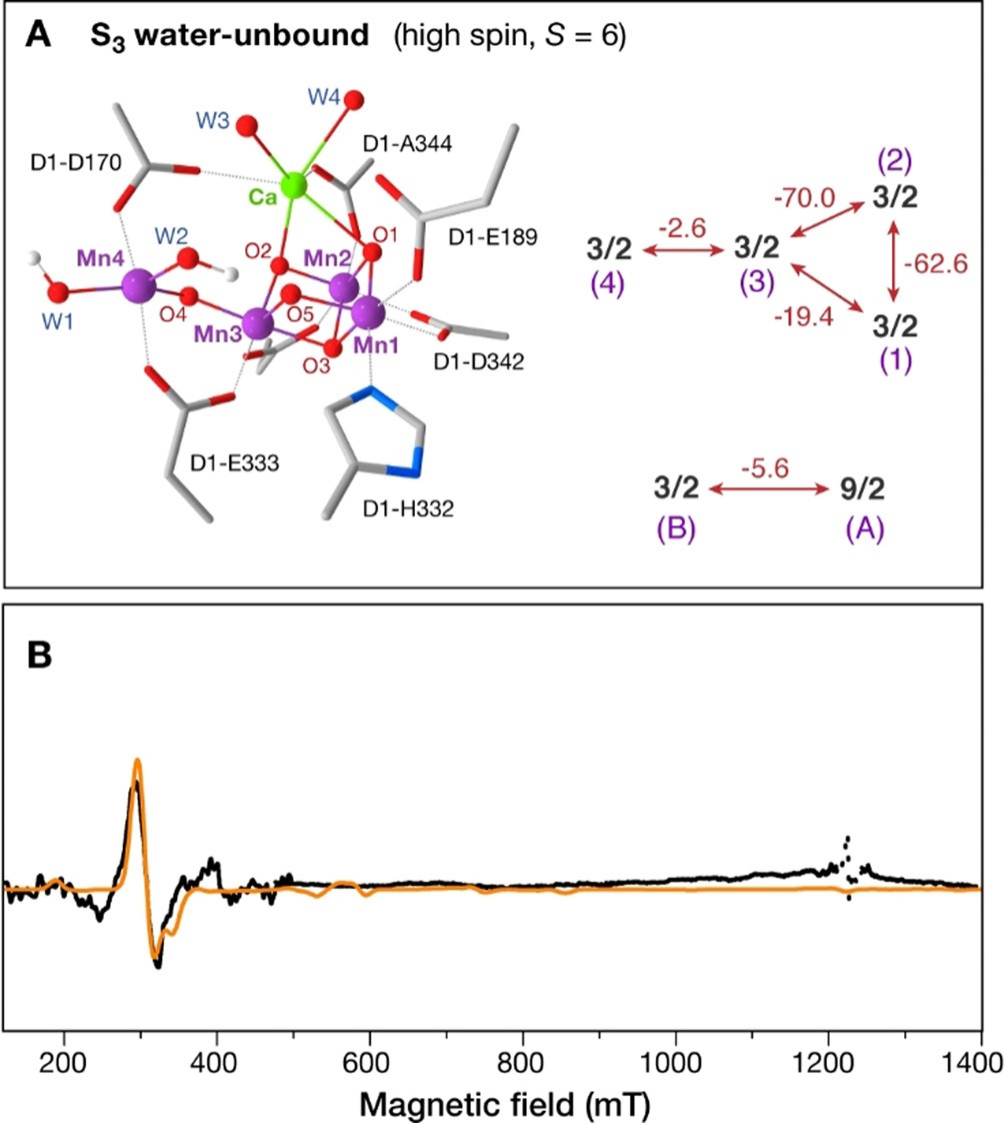
A) Geometry of the “water‐unbound” conformation of the S_3_ state with a 5‐coordinated Mn4(IV) ion reported by quantum chemical simulations,^[30]^ with the computed exchange coupling constants leading to the high‐spin ground state of *S=*6. The ladder of the four lowest spin states of the system can be reproduced by an effective ferromagnetic exchange coupling of −5.6 cm^−1^ in a two‐spin system of *S*_A_=9/2 and *S*_B_=3/2 that stands for the “3+1” magnetic representation of the OEC (see also Figure S5). B) Simulation (orange curve) of the Q‐band EPR spectrum (black curve) attributed to the *S=*6 state in MeOH‐containing samples by assuming anisotropic ferromagnetic exchange between the *S*_A_=9/2 and the *S*_B_=3/2 spins, and the spin Hamiltonian parameters *D*_9/2_=+0.273 cm^−1^, *D*_3/2_=+2.14 cm^−1^, (*E*/*D*)_9/2_=(*E*/*D*)_3/2_=0.14, *g*_9/2_=1.98, *g*_3/2_=1.98, *J_xx_*=*J_yy_*=−1.382 cm^−1^, *J_zz_*=−14.036 cm^−1^.

A plausible all‐Mn^IV^ S_3_ conformation of the OEC that has a *S=*6 ground state has been reported by Retegan et al.^[30]^ This “water‐unbound” computational model, depicted in Figure 4, resembles the geometry attributed to the high‐*g* isomeric form of the preceding S_2_ state (Figure 1), featuring a Mn_3_CaO_4_ subunit attached to a pendant five‐coordinated Mn^IV^ ion. Computed isotropic exchange coupling constants for this model show ferromagnetic interaction between Mn3 and the coordinatively unsaturated Mn4 (Figure 4).^[30]^ This exchange coupling, suggested to be key in determining the total spin state of the cluster, cannot become antiferromagnetic because the distorted geometry of Mn4 abolishes super‐exchange over the only possible O4 pathway, leading to the highest possible spin *S=*6 for the ground state. The fact that the water‐unbound model of Figure 4 explains both the high‐spin *S=*6 configuration of the S_3_ state and the methanol‐induced attenuation of substrate binding leads us to tentatively assign the *S=*6 component identified in the present study to this conformation. Under this assumption, the EPR observations described above suggest that the water‐unbound configuration constitutes the majority of the physiological S_3_ state and almost the complete population of the MeOH‐treated S_3_ state in spinach PSII.

The local zero field splitting value of the five‐coordinated Mn4(IV) ion was computed^[30]^ to be unexpectedly high for a Mn^IV^ ion, *D*_4_=+2.14 cm^−1^, which is however in line with synthetic analogs,^[31]^ while *D_i_* values for the other Mn ions are small and typical of six‐coordinated Mn^IV^.^[32]^ Based on the local second order zero field splitting values of the Mn ions we investigated the origin of the effective zero field splitting of the two spin configurations described in the present EPR investigation. While the value of |*D*|=0.179 cm^−1^ for the *S=*3 multiplet is easily accounted for by the local contributions of four octahedral Mn^IV^ ions, in the case of the *S=*6 configuration the local second order zero field splitting contributions do not constitute the unique origin for the effective zero field splitting *D*≈1.5 cm^−1^. To understand this crucial aspect of the system it is necessary to analyze its magnetic structure in finer detail. Utilizing the “3+1” motif of the OEC, the isotropic low‐energy spectrum of the four‐spin system that describes the water‐unbound model,^[30]^ that is, the ladder of lowest energy spin states with *S=*6, 5, 4, and 3, can be exactly reproduced with an effective isotropic ferromagnetic *J*_eff_ of −5.6 cm^−1^ between the fictitious spins *S*_A_=9/2 and *S*_B_=3/2 that represent the trimer (Mn1‐Mn2‐Mn3) and monomer (Mn4) manganese subunits (Figure S4). Therefore, the two‐spin model serves as proxy of the four‐spin system for the low‐energy spin states. Crucially, the weak effective ferromagnetic coupling is of the same magnitude as the local anisotropy of Mn4, therefore the usual simplified assumptions regarding the strong exchange limit do not apply and cannot justify the high effective *D* value. Previous studies of exchange coupled clusters highlighted the impact of the exchange coupling anisotropy on the splitting of spin state multiplets at zero magnetic field.[^33^, ^34^] Therefore, in our case the four ferromagnetically anisotropic exchange coupled Mn^IV^ ions should explain the relatively large effective zero field splitting.

In order to investigate the contribution of anisotropic exchange to the effective *D* of the *S=*6 multiplet, additional EPR simulations were performed by introducing anisotropic exchange interaction between the trimanganese unit with *S*_A_=9/2 and the outer five‐coordinated Mn4(IV) with *S*_B_=3/2. Figure 4 shows that the theoretical spectrum obtained on the assumption of anisotropic exchange coupling between *S*_A_=9/2 and *S*_B_=3/2 with *D*_9/2_=+0.273 cm^−1^, *D*_3/2_=+2.14 cm^−1^, (*E*/*D*)_9/2_=(*E*/*D*)_3/2_=0.14, *g*_9/2_=1.98, *g*_3/2_=1.98, *J_xx_*=*J_yy_*=−1.382 cm^−1^, *J_zz_*=−14.036 cm^−1^ reproduces very well the Q‐band EPR spectrum attributed to the *S=*6 configuration. Small variations of spin Hamiltonian parameters reproduce the EPR spectrum almost equally well, but only under the condition of anisotropic exchange.

In conclusion, the effective zero field splitting of the *S=*6 state multiplet originates both from the local second order zero field splitting terms of the Mn ions *and* from anisotropic exchange interactions. Therefore, the present results and analysis strongly support the water‐unbound model shown in Figure 4 as the origin of the *S=*6 signal in the S_3_ state of spinach PSII.

### Implications for the Mechanism of Water Oxidation

Matching the two observed EPR signals of *S=*3 and *S=*6 with specific geometric configurations of the OEC cluster has important implications for understanding the nature of the S_3_ state itself and the catalytic progression of water oxidation in higher plants, and possibly across oxygenic photosynthetic organisms. The results establish that in spinach PSII the majority of the S_3_ state exists physiologically as a mixture of water‐unbound and water‐bound conformations. This must be given serious consideration in the analysis of other experimental observations. It also calls to question the validity of “single‐component” structural interpretations derived from crystallographic studies that are so far unable to resolve state‐specific structural heterogeneity and show irregularities in the definition of the central O atom positions.[^1^, ^2^, ^3^] These may well arise from mixtures of the different S_3_ forms discussed here. Furthermore, the almost complete arrest of water binding in methanol‐treated samples without inhibition of Mn oxidation establishes that Mn oxidation in the S_2_→S_3_ transition strictly precedes and is completed independently from water binding. Water binding occurs at a component of the S_3_ state proper, that is, after reduction of the Y_Z_^.^ radical. This suggests that the conformation represented by the *S=*6 EPR signal corresponds to the S_3_ population formed under normal catalytic progression, disfavoring early water binding^[35]^ in the S_2_ state.

Both Mn1 and Mn4 are discussed as possible sites of water binding in the S_2_→S_3_ transition because they can offer a coordination site in the S_2_ state. Observations relating to methanol and ammonia interaction with the OEC in the S_2_ state support the idea that water binds externally to a 5‐coordinated Mn4 site,[^30^, ^36^] whereas other alternatives include the shift of a Ca‐bound or a second‐sphere water molecule embedded in the surrounding hydrogen‐bonded water network to either of the terminal Mn ions.[^37^, ^38^, ^39^] Pulse ENDOR studies on spinach PSII using ^13^C‐ or ^2^H‐ labeled methanol are consistent with MeOH positioned either close to Mn4 or close to Ca^2+^ in the S_2_ state^[9]^ and hence water delivery might be arrested by MeOH from either direction. Attribution of the *S=*6 signal to a species with a five‐coordinated Mn4 ion supports Mn4 as the site of initial water binding in the S_3_ state.

The attribution of the *S=*6 water‐unbound component to a majority S_3_ species in spinach PSII implies an enthalpic barrier to water binding itself, if it occurs directly at Mn4, or due to additional rearrangements and proton translocations required to form the “oxo‐hydroxo” *S=*3 component. The latter multi‐step pathway may involve several water‐bound forms,^[40]^ possibly all with the same *S=*3 spin state. The identity of atoms that proceed from a water‐bound S_3_ form to create the O−O bond in the final catalytic transition via radical coupling[^41^, ^42^] would be different depending on whether the substrate is delivered internally or externally to Mn4, O5‐W2 being the most likely assignment in the case of external water binding.^[43]^

**Figure 5:**
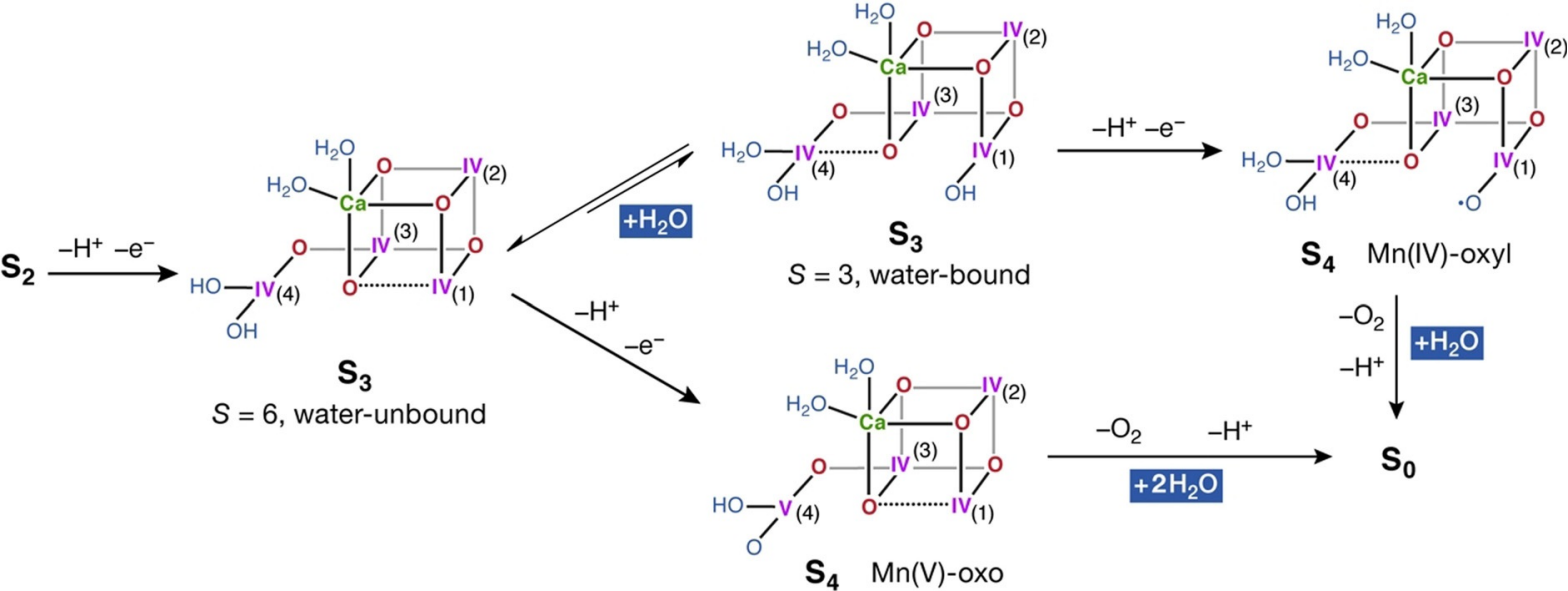
Possible mechanistic pathways from the *S=*6 and *S=*3 components of the S_3_ state. The *S=*6 water‐unbound form may be considered the precursor to a catalytically active *S=*3 water‐bound form that is oxidized to an S_4_ Mn^IV^‐oxyl intermediate (pathway at the top),[^41^, ^42^] supporting radical‐type O–O coupling. The identity of substrates would depend both on the precise mechanism of O−O bond formation and on the binding site and position of the newly inserted water. Each transformation is presumably multi‐step. Indicated protonation states of terminal ligands are adopted from literature suggestions and are not constrained by the present work. Alternatively, the *S=*6 form may be catalytically active itself (pathway at the bottom) and give rise to a 5‐coordinated S_4_ Mn^V^‐oxo intermediate,^[44]^ allowing for nucleophilic coupling.^[44]^ In this case, water binding is deferred and O−O bond formation is only possible between oxygen atoms pre‐existing in the resting state. In the latter scenario the OEC stores all four water‐oxidizing equivalents on Mn ions prior to dioxygen evolution and binds new water molecules only between the S_4_ and S_0_ states, to make them available as substrates already upon initiation of the next catalytic cycle.

However, an alternative scenario gains weight from the present results. The practically complete conversion of the higher‐plant S_3_ state into a water‐unbound form via methanol treatment does not inhibit oxygen evolution *per se*, because flashing of the water‐unbound S_3_ state still enables progression to S_0_. This leaves open the question whether water binding in the S_3_ state is at all required for the final S_3_→S_4_ catalytic step, and hence the question which component of the S_3_ state is catalytically active. Krewald et al. suggested that water binding is not obligatory for final oxidation of the OEC cluster to the S_4_ state.^[44]^ The latter can be formulated as containing either a Mn^IV^‐oxyl group,[^41^, ^42^] if advancement occurs from the oxo‐hydroxo S_3_ form, or a 5‐coordinated Mn^V^‐oxo group,^[44]^ if advancement occurs from a water‐unbound S_3_ population. Further experimental studies are clearly required to resolve the intermediates of the S_3_→S_4_ transition, but the above considerations support the intriguing possibility that all productive catalytic transitions in biological water oxidation past the resting S_1_ state may occur physiologically via distinct configurations of the metastable heterogeneous S_2_ and S_3_ states with pre‐bound substrates, without requiring additional water binding up until dioxygen evolution and reconstitution of the S_0_ state (Figure 5).

## Conclusion

EPR studies of the S_3_ oxidation state of the OEC at X‐ and Q‐band in intact spinach PSII and in methanol‐treated PSII preparations help to resolve the spin and structural heterogeneity originating from different forms of the OEC in the crucial S_3_ state. The results show that in intact PSII the S_3_ EPR spectra can be described by a combination of two sets of spin Hamiltonian parameters, one corresponding to a known intermediate‐spin *S=*3 species and the other to a previously uncharacterized high‐spin *S=*6 form. In contrast, the spectra of the S_3_ state in methanol‐treated PSII preparations can be simulated with a unique set of spin Hamiltonian parameters that correspond to the *S=*6 form because formation of the intermediate‐spin conformation is inhibited. This high‐spin component is characterized by a high effective zero field splitting parameter that renders it unobservable in X‐band and indicates a significantly different geometric and electronic structure compared to the *S=*3 form that has been attributed to an “oxo‐hydroxo” type of geometry. In contrast to the *S=*3 multiplet the splitting of the *S=*6 multiplet at zero field originates both from the local second order zero field splitting contributions of the Mn ions and from anisotropic exchange interactions.

The coexistence of the *S=*3 and *S=*6 configurations of S_3_ ascertains the presence of structurally distinct components in the sense of water‐unbound (*S=*6) and water‐bound (*S=*3) forms, which so far have not been resolved by structural methods of investigation such as protein crystallography. The results confirm that Mn oxidation in the S_2_→S_3_ transition is independent of water binding. Both components of the S_3_ state can in principle advance to the oxygen‐evolving S_4_ state, therefore it is possible to formulate distinct structural and electronic configurations of the cluster that support either radical‐type or nucleophilic O‐O coupling, with fundamental consequences for understanding the nature of redox progression ‐charging versus catalysis‐ within the catalytic cycle. The intrinsic heterogeneity of the S_3_ state uncovered in the present work has important implications for correctly evaluating the information derived from less discriminating experimental approaches and for directing future mechanistic investigations into the most critical final S_3_→S_4_ step that leads to dioxygen evolution.

## Conflict of interest

The authors declare no conflict of interest.

## Supporting information

As a service to our authors and readers, this journal provides supporting information supplied by the authors. Such materials are peer reviewed and may be re‐organized for online delivery, but are not copy‐edited or typeset. Technical support issues arising from supporting information (other than missing files) should be addressed to the authors.

Supplementary
