# Supplementary material for: Arrested Substrate Binding Resolves Catalytic Intermediates in Higher‐Plant Water Oxidation
Source: Angew Chem Int Ed Engl. 2020 Dec 10;60(6):3156–62. doi: 10.1002/anie.202012304 (PMC7898718; doi:10.1002/anie.202012304)
Supplement: Supplementary file 1 — Supplementary [file ANIE-60-3156-s001.pdf]

Angewandte Chemie
Eine Zeitschrift der Gesellschaft Deutscher Chemiker

Supporting Information

Arrested Substrate Binding Resolves Catalytic Intermediates in Higher-Plant Water Oxidation

Georgia Zahariou, Nikolaos Ioannidis, Yiannis Sanakis, and Dimitrios A. Pantazis**

anie_202012304_sm_miscellaneous_information.pdf

PSII sample isolation

PSII-enriched thylakoid membranes were isolated from spinach by standard procedures.[1-2] Samples for EPR measurements were suspended in a pH=6.5 buffer containing 0.4 M sucrose, 15 mM NaCl, 40 mM MES, pH 6.5, at about 6–8 mg chl/mL and stored in liquid nitrogen until use. Methanol treated samples were resuspended and washed in the above buffer containing 5% (v/v) methanol. All samples prior to the EPR experiments were given a pre-flash at -10 °C and were subsequently dark adapted for 40 min at 0–4 °C. This poised all centers to the S_1 state. The samples were subsequently supplemented with 1mM of an exogenous electron acceptor, either di-chloro-p-benzoquinone, DCBQ, or Phenyl-p-benzoquinone, PpBQ.[3]

Illumination conditions

A studio photographic flash unit, Elinchrom Style RX 1200, with variable flash power up to 1200 Ws, and pulse duration (half width at half height) of 1–2 ms was used for flash excitation of the samples. The S_3 state was formed by two flash illumination on the S_1 state at 253 K.

EPR measurements

EPR measurements were obtained with an extensively upgraded former Bruker ER-200D spectrometer interfaced to a personal computer and equipped with an Oxford ESR 900 cryostat, an Anritsu MF76A frequency counter, a Bruker 035M NMR gaussmeter and a SR830 digital lock-in amplifier by Stanford Research. The perpendicular 4102ST and the dual mode 4116DM cavities were used at X-band for the perpendicular and the parallel mode, respectively, while at Q-band the perpendicular ER5106QT cavity was used. The experimental conditions of the EPR measurements are the following. For the perpendicular mode X-band experiments: microwave frequency: 9.41 GHz, microwave power: 32 mW, modulation amplitude: 25 Gpp, modulation frequency: 100 kHz, T=10 K. For the parallel mode X-band measurements: microwave frequency: 9.31 GHz, microwave power: 126 mW, modulation amplitude: 10 Gpp, modulation

frequency: 100 kHz, T=10 K. For the perpendicular mode Q-band experiments: microwave frequency: 34.55 GHz, microwave power: 126 mW, modulation amplitude: 25 Gpp, modulation frequency: 100 kHz, T=10 K.

Simulation of the EPR spectra

The theoretical simulation of the EPR spectrum was performed with *EasySpin*.[4] For the approximation of an isolated spin state, the following spin Hamiltonian was used:

$$\hat{H}_0 = g\beta\vec{B}\cdot\vec{S} + D\left[\vec{S}_z^2 - \frac{1}{3}S(S+1)\right] + E\left(\vec{S}_x^2 - \vec{S}_y^2\right) \tag{S1}$$

In equation S1 the first term is the Zeeman interaction. The second and third terms represent the zero field interaction, were *D* and *E* are the axial and rhombic zero field splitting parameters, respectively.

For the detailed investigation on the S_3 possible spin states of the MeOH containing spinach PSII preparations, apart from the second order zero field splitting parameters, additional fourth-order zero field splitting parameters were taken into account and the following more detailed spin Hamiltonian was used.

$$\hat{H}_2 = \hat{H}_0 + \frac{a}{6}\left[\vec{S}_x^4 + \vec{S}_y^4 + \vec{S}_z^4 - \frac{1}{5}S(S+1)(3S^2+3S-1)\right]$$
$$+\frac{F}{180}\left[35\vec{S}_z^4 - 30S(S+1)\vec{S}_z^2 + 25\vec{S}_z^2 - 6S(S+1) + 3S^2(S+1)^2\right] \tag{S2}$$

For the approach of the two exchange coupled spins, the spin Hamiltonian shown in equation S3, which contains the exchange interaction terms was used:

$$\hat{H}_1 = \hat{H}_0 + J_{xx}\vec{S}_{1x}\cdot\vec{S}_{2x} + J_{yy}\vec{S}_{1y}\cdot\vec{S}_{2y} + J_{zz}\vec{S}_{1z}\cdot\vec{S}_{2z} \tag{S3}$$

Theoretical EPR spectra obtained by assuming isolated spin states of *S*=1–6

In order to determine the spin configuration of the S_3 state that gives rise to the low-field EPR feature at Q-band, we performed a detailed theoretical investigation of the S_3 EPR spectrum in methanol-containing PSII membranes. First of all, in Figure S1 we show the superposition of the S_3 experimental EPR spectrum at Q-band with the simulated one obtained by using a unique spin configuration of S=3, g=2, $|D|$=0.179 cm^{-1}, E/D=0.28, σ_D=0.018 cm^{-1}. It is obvious that the theoretical spectrum that results from the aforementioned parameters that were fitted to the X-band spectrum cannot match several features of the Q-band EPR spectrum. This indicates that an additional spin configuration is necessary to fit the experimental spectrum.

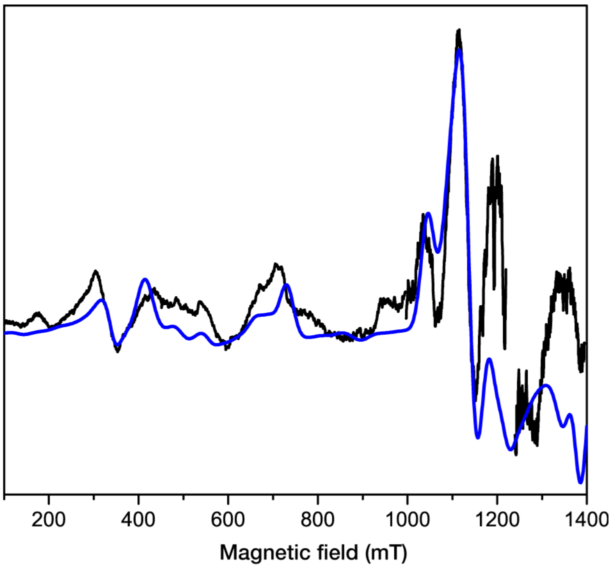

Figure S1. Superposition of the S_3 experimental EPR spectrum at Q-band (black trace) with the simulated spectrum (blue) using a unique spin configuration of S=3, g=2, $|D|$=0.179 cm^{-1}, E/D=0.28, σ_D=0.018 cm^{-1}.

Considering that the electronic distribution of the Mn_4CaO_5 in the S_3 state is Mn(IV)$_4$[5] we investigated which of the possible spin values of S = 1, 2, 3, 4, 5, or 6 matches better the corresponding experimental feature at low-field region (Panel B of Figure 2). To achieve proper comparison for all of the above possible spin states, we used D and E/D values of spin Hamiltonian, so that the corresponding EPR absorptions take place at the low field region. For PSII preparations in the presence of MeOH the S_3 EPR spectrum at Q-band consists of a

derivative centered at ca. 300 mT, together with the low intense EPR features around 200 mT and 400 mT, as shown in Figure S2. By comparing the simulation spectra obtained by assuming S=1 and S=6 (traces magenta and blue of Figure S2, respectively) we observe that both theoretical curves can reproduce the derivative at ~300 mT. However, apart from this derivative the additional EPR features cannot be described by assuming a spin of S=1. Instead, these features can be sufficiently described by assuming the S=6 configuration. Overall, among the various theoretical spectra, the one obtained by assuming an isolated spin state with S=6 and the parameters of g=1.98, D=+1.523 cm^{-1}, E/D=0.14 describes best the experimental EPR features at g_{eff}~8. By using the aforementioned parameters, the theoretical spectrum at X-band presents no EPR signal, in agreement with the experimental data and with previous reports.[6]

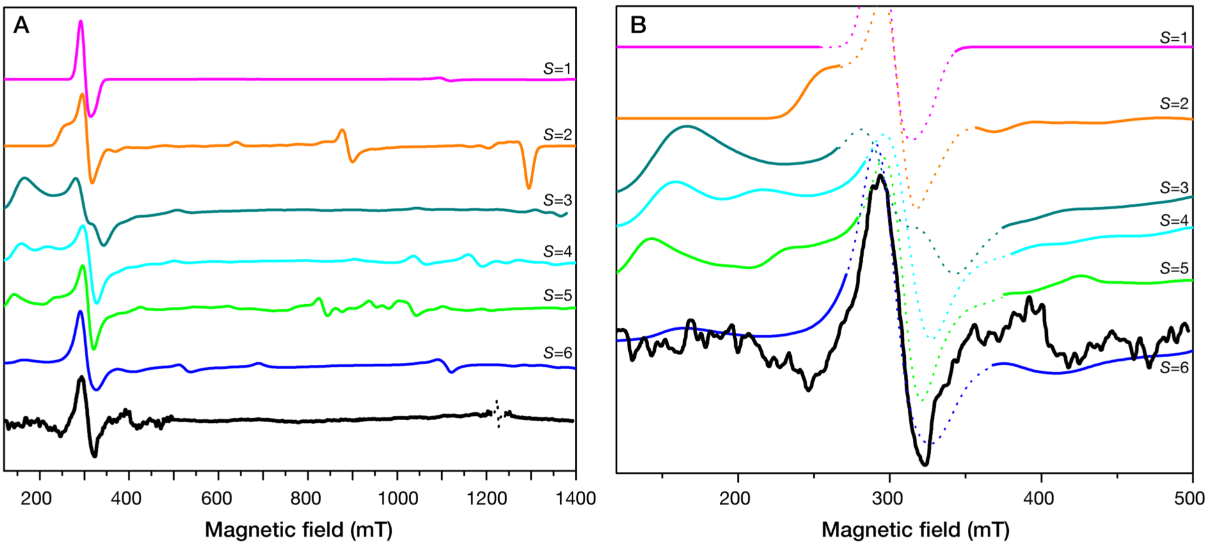

Figure S2. Comparison of the experimental EPR spectrum of the S_3 state at Q-band in MeOH-containing PSII with various theoretical spectra obtained by assuming integer spins of S=1–6. Panel A: Experimental spectrum at Q-band (black curve) and various theoretical spectra obtained by assuming S=1, g=2, D=±0.76 cm^{-1}, E/D=0.33 (magenta), S=2, g=2, D=±1.02 cm^{-1}, E/D=0.175 (orange), S=3, g=2, D=±0.795 cm^{-1}, E/D=0.33 (grey), S=4, g=2, D=±0.726 cm^{-1}, E/D=0.196 (cyan), S=5, g=2, D=±0.652 cm^{-1}, E/D=0.13 (green), S=6, g=1.98, D=+1.523 cm^{-1}, E/D=0.14 (blue). Panel B: Magnification of the low field region.

It should be noted that recent simulations on S_3 cyanobacterial PSII showed that apart from the second order zero field splitting parameters, additional higher-order zero field splitting parameters were needed to reproduce the experimental EPR spectrum.[7] Therefore, fourth order zero field splitting parameters described in equation S2 were taken into account for the present detailed investigation on the simulation of the experimental EPR spectrum with spin states of *S*=1–5. However, the use of these additional terms for these spin values fails to reproduce the S_3 experimental EPR spectrum.

Energy level diagrams at Q-band and respective resonance transitions for the *S*=6 configuration

In Figure S3 we present the energy levels diagrams with the resonance transitions that explain the S_3 EPR signals of the *S*=6 and *S*=3 configurations.

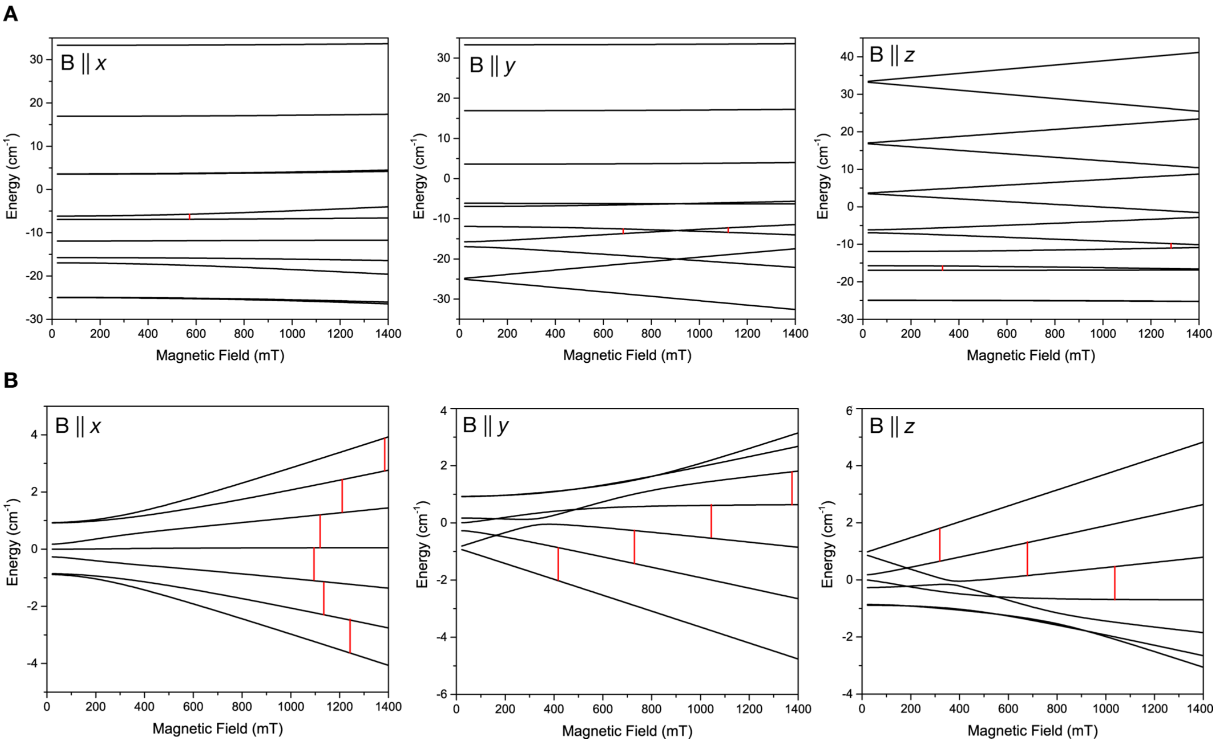

Figure S3. Energy level diagrams for the *S*=6 (A) and *S*=3 (B) EPR signals of the S_3 state and the respective EPR transitions (red lines). The three panels in each row correspond to the B field aligned along the three principal axes.

Origin of the zero field splitting for the *S*=3 and *S*=6 ground state multiplets of the S_3 oxidation state of the OEC

In exchange coupled clusters the zero field splitting parameter, *D*, of the various spin multiplets originates from the local zero field splitting parameter D_i, while there are cases where anisotropic exchange interactions have been also considered.[8-9] In the former case, *D* is given by the relationship[10]:

$$D = \sum d_i D_i \quad \text{(S4)}$$

where

$$d_i = f(S_T, S_i) \quad \text{(S5)}$$

Taking into account the previously reported nature of the spin-spin interactions within the OEC in the S_3 state that give rise to the ground states of *S*=3 and *S*=6, as well as the local second order zero field splitting terms,[11-12] we applied equation S4 to examine whether the local contributions constitute the exclusive origin for the zero field splitting of the respective multiplets.

The most supported geometric structure of the S_3 state that leads to the ground spin configuration of *S*=3 contains four octahedral Mn(IV) ions.[5, 13-14] A structure of the S_3 state that was shown to have a ground-state spin of *S*=6 while still containing only Mn(IV) ions consists of three octahedral Mn(IV) ions of the Mn_3CaO_4 unit and an outer five-coordinated Mn4(IV),[11] i.e. a water-unbound form of the S_3 state. D_i values of the octahedral Mn(IV) ions are typical for this coordination geometry,[12] while that of the five-coordinated Mn4(IV) ion was computed to be 2.14 cm^{-1} from DFT.[11] We adopt this structure as the most suitable candidate for the *S*=6 component of the S_3 state because its properties are consistent with the concept of methanol-induced arrest of water binding and because its properties are consistent with the present spectroscopic observations.

Based on the above, the application of the equation S4 for the magnetic properties of the geometric structures of the ground spin *S*=3 multiplet gives a range for the effective *D* of

$|D_{S=3}|=\{0.054\ cm^{-1} - 1.23\ cm^{-1}\}$. The parameter of $|D|=0.179\ cm^{-1}$ that arose from our present simulated analysis of the $S=3$ configuration lies in this range, indicating that the splitting of the $S=3$ ground spin multiplet at zero field presumably originates exclusively from the local second order zero field splitting terms of the four exchanged-coupled Mn(IV) ions.

Regarding the $S=6$ case, the use of the equation S4 for the ferromagnetically coupled Mn(IV) ions gives a value for the effective $|D_{S=6|}$ between the range of $0.11\ cm^{-1}$and $0.4\ cm^{-1}$. In contrast, our simulation study of the $S=6$ configuration reveals a zero field splitting parameter of $D=+1.523\ cm^{-1}$, a value much higher than the maximum one of the above range. This strongly indicates that the local second order zero field splitting terms of the four individual Mn(IV) ions do not contribute exclusively to the effective zero field splitting. Thus, the additional effect of anisotropic exchange interactions between the Mn(IV) ions must also be considered as a contributor to the total D_{eff}, as explained in the main text.

Exchange coupling in the *S*=3 and *S*=6 forms of the S_3 state

Figure S4 depicts the exchange coupling constants and part of the resulting Heisenberg spin ladder as reported by quantum chemical simulations on the intermediate-spin (*S*=3), oxo-hydroxo S_3 conformation.[5] Figure S5 depicts the same information[11] for the high-spin (*S*=6) water-unbound S_3 conformation. The low-energy part of the Heisenberg ladder of the high-spin form can be simulated in the "3+1" representation of the OEC cluster with effective spins S_A = 9/2 (for the trinuclear part) and S_B= 3/2 (for the five-coordinated Mn4(IV) ion) and an effective exchange coupling that is approximately double in magnitude to the $J_{Mn3–Mn4}$ coupling of the 4-spin model.

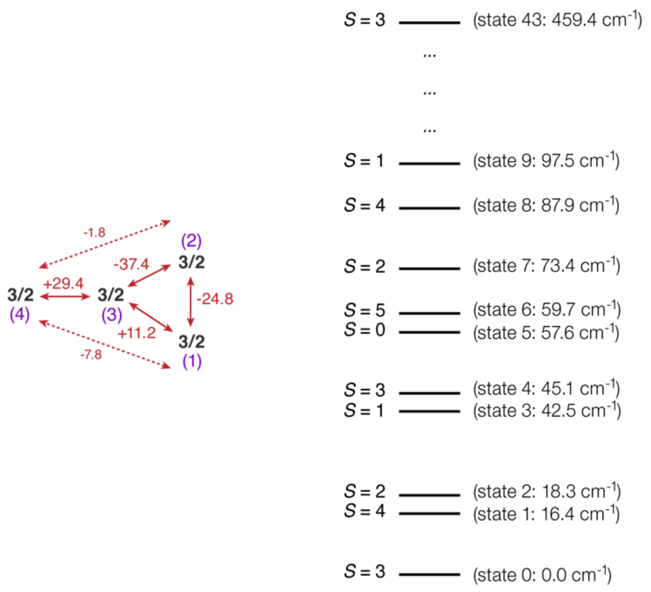

Figure S4. Exchange coupling in the $S = 3$ form of the S_3 state.The scheme on the left depicts computed isotropic exchange coupling constants for the water-bound (oxo–hydroxo) form of the S_3 state. On the right a few of the resulting spin levels (within 100 cm^{-1} of the ground state) are schematically depicted. This spin ladder configuration is used in order to estimate the thermal occupation of the $S = 3$ ground state at the conditions of the EPR experiments (10K).

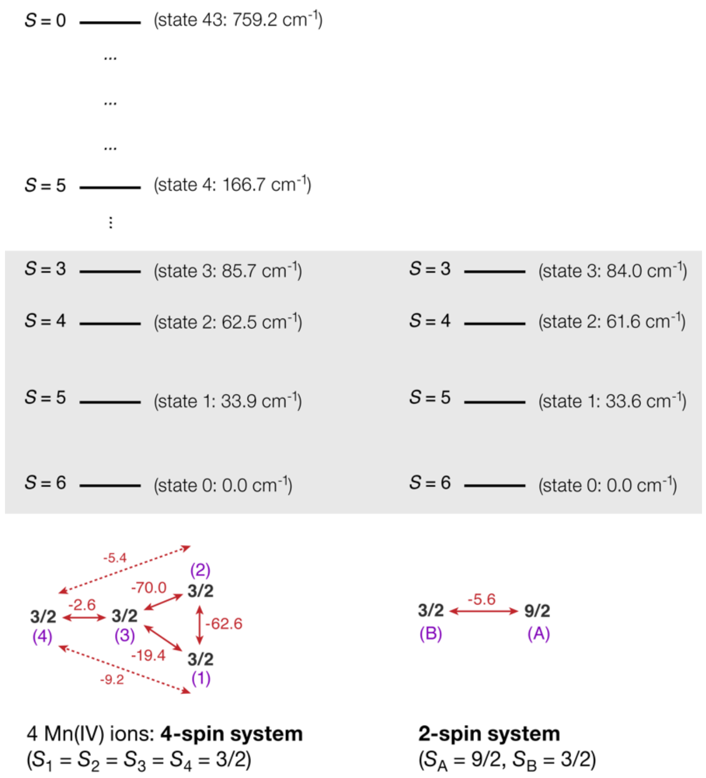

Figure S5. Reduction of the 4-spin to an effective 2-spin representation. The scheme on the left depicts the computed isotropic exchange coupling constants for the water-unbound S_3 state identified with the S = 6 signal (the exchange coupling values are taken from Retegan et al.[11] and converted to conform with our convention for the Heisenberg exchange Hamiltonian, $H = +\Sigma J_{ij}S_iS_j$, where negative J values denote ferromagnetic interaction). The Heisenberg spin ladder of the 4-spin system comprises 44 spin states. The four lowest states can be modelled with an effective 2-spin system shown on the rightthat represents the trinuclear and mononuclear Mn subunits of the cluster in this configuration. An effective exchange coupling of -5.6 cm^{-1} almost exactly reproduces the lowest spin levels of the 4-spin system. This spin ladder configuration is used in order to estimate the thermal occupation of the $S = 6$ ground state at the conditions of the EPR experiments (10K).

References

[1] D. A. Berthold, G. T. Babcock, C. F. Yocum, *FEBS Lett.* **1981**, *134*, 231-234.
[2] R. C. Ford, M. C. W. Evans, *FEBS Lett.* **1983**, *160*, 159-164.
[3] V. Petrouleas, B. A. Diner, *Biochim. Biophys. Acta Bioenerg.* **1987**, *893*, 126-137.
[4] S. Stoll, A. Schweiger, *J. Magn. Reson.* **2006**, *178*, 42-55.
[5] N. Cox, M. Retegan, F. Neese, D. A. Pantazis, A. Boussac, W. Lubitz, *Science* **2014**, *345*, 804-808.
[6] T. Matsukawa, H. Mino, D. Yoneda, A. Kawamori, *Biochemistry* **1999**, *38*, 4072-4077.
[7] M. Chrysina, E. Heyno, Y. Kutin, M. Reus, H. Nilsson, M. M. Nowaczyk, S. DeBeer, F. Neese, J. Messinger, W. Lubitz, N. Cox, *Proc. Natl. Acad. Sci. U. S. A.* **2019**, *116*, 16841.
[8] K. R. Vignesh, S. K. Langley, C. J. Gartshore, I. Borilović, C. M. Forsyth, G. Rajaraman, K. S. Murray, *Dalton Trans.* **2018**, *47*, 11820-11833.
[9] Y. Sanakis, A. L. Macedo, I. Moura, J. J. G. Moura, V. Papaefthymiou, E. Münck, *J. Am. Chem. Soc.* **2000**, *122*, 11855-11863.
[10] A. Bencini, D. Gatteschi, *EPR of Exchange Coupled Systems*, Springer Verlag, Berlin, **1990**, p. 287.
[11] M. Retegan, V. Krewald, F. Mamedov, F. Neese, W. Lubitz, N. Cox, D. A. Pantazis, *Chem. Sci.* **2016**, *7*, 72-84.
[12] M. Zlatar, M. Gruden, O. Y. Vassilyeva, E. A. Buvaylo, A. N. Ponomarev, S. A. Zvyagin, J. Wosnitza, J. Krzystek, P. Garcia-Fernandez, C. Duboc, *Inorg. Chem.* **2016**, *55*, 1192-1201.
[13] V. Krewald, M. Retegan, N. Cox, J. Messinger, W. Lubitz, S. DeBeer, F. Neese, D. A. Pantazis, *Chem. Sci.* **2015**, *6*, 1676-1695.
[14] N. J. Beal, T. A. Corry, P. J. O'Malley, *J. Phys. Chem. B* **2018**, *122*, 1394-1407.
